# Editorial: Pharmacological and non-pharmacological therapy for obesity and diabetes - volume II

**DOI:** 10.3389/fendo.2023.1252536

**Published:** 2023-10-20

**Authors:** Alexandre Gabarra Oliveira, Bruno Melo Carvalho, Guilherme Zweig Rocha

**Affiliations:** ^1^ Department of Physical Education, Bioscience Institute, São Paulo State University (UNESP), Rio Claro, São Paulo, Brazil; ^2^ Institute of Biological Sciences, University of Pernambuco (UPE), Recife, Pernambuco, Brazil; ^3^ Department of Internal Medicine, School of Medical Sciences, University of Campinas (UNICAMP), Campinas, São Paulo, Brazil

**Keywords:** obesity, diabetes, morbidity, cardiovascular risk, insulin resistance

Obesity is one of the most threatening diseases due to its medium to long term consequences and complications, such as type 2 diabetes, cardiovascular diseases, nonalcoholic fatty liver disease, several types of cancer and others (1–4). Interrelationships between insulin resistance and chronic subclinical inflammation adversely affect cellular metabolic functions, homeostasis with generalised metabolic derangement. It is also associated with other phenomena such as beta-cell dysfunction, inflammasome activation, oxidative stress and endoplasmic reticulum stress (ER stress), which also supports the insulin resistance development and maintenance.

Together, these health conditions inflict a considerable burden on individuals, society, and on the economy, through greater public health costs, morbidity and mortality. In this Research Topic, Genua et al. have shown that obesity is also frequent in people with type 1 diabetes, and that these patients also have a higher prevalence of other cardiovascular risk factors. In line with this connection between obesity and cardiovascular risk factors, Liu et al. showed here that in a meta-analysis regarding the use of SGLT2 inhibitors, a widely used drug class for the treatment of cardiovascular diseases and type 2 diabetes, also has some effects in decreasing visceral and subcutaneous adipose tissue, as well as body weight and triglycerides in type 2 diabetes patient. Additionally, another method that also modestly reduces body weight, fat tissue and triglyceride is the long-term acupoint stimulation, a modified acupuncture technique, that showed some interesting results in a randomized controlled trial, as shown here by Dai et al. It is also suggested by Yang et al., in an interesting review, that the use of plant secondary metabolites, such as flavonoids, alkaloids, terpenoids, resveratrol, lipoic acid and others show lipolytic activities and could be beneficial for weight control and the reduction of obesity related risks. Several of these compounds act by the activation of Hormone Sensitive Lipase (HSL), which increases the fatty acids disposal, and AMPK, which induces lipid β-oxidation.

Insulin resistance is often accompanied by a compensatory elevation in insulin production and secretion that is supported by both hypertrophy and hyperplasia of pancreatic beta cells (5, 6). Although, during the T2DM onset such compensatory mechanism is missing because many beta-cells are dysfunctional as a consequence of increased oxidative stress among others (7, 8). Besides, obesity also induces important alterations in islet microenvironment organization such as rising pancreatic stellate cells (9). It is already known that the use of TUDCA (tauroursodeoxycholic acid) positively impacts on beta-cell function in obese models (10), and novel features of this molecule show interesting activity on adipose tissue, through G protein-coupled bile acid receptor 1 (TGR5) and farnesoid X Receptor (FXR) receptors activation (Freitas et al.).

On the other hand, N-acetyl-L-cysteine (NAC) may attenuate oxidative stress and insulin resistance due to its anti-inflammatory and antioxidant effects (11). Indeed, in the current Research Topic Schuurman et al. have demonstrated that NAC treatment is able to reduce both beta-cell oxidative stress and pancreatic stellate cell (PaSC) activation, along with a normalization of beta-cell mass and size in high-fat diet-induced diabetic mice. They also observed that there is an optimal timing and dosage. Thus, this study extended the current knowledge by highlighting that an antioxidant treatment such as NAC may have protective effects on beta-cell health in obesity.

It is well-known that the chronic inflammation present in obesity has an important relation with the hypoxia observed in adipose tissue expansion. In fact, hypoxia may have a pivotal role in the development of adipose tissue dysfunction through HIF-1α activity (12, 13). In this regard, this Research Topic brings a study of Wu et al. that deeply addressed this point since they demonstrated that nicotinamide mononucleotide effectively reduced fibrosis induced by HIF-1α in adipose tissue of mice placed in a hypobaric chamber for 4 weeks. They also saw that nicotinamide mononucleotide restores NAD/SIRT1 axis. These findings shed light on the nicotinamide mononucleotide as a regulator of adipose tissue hypoxia.

We also highlight that obese patients are also susceptible for nutritional deficiency, which is a low-observed and -investigated component in obesity, as indicated by Shadai et al. (Sadhai et al.), in a group of south-Africans scheduled for bariatric surgery. The most common nutritional deficiency observed in obese people who will undergo metabolic surgery was vitamin D (57%), followed by iron and folate deficiency (44% and 18%, respectively). They also suggest including these items on the preoperative screening of the patients and to include them in a longitudinal surveillance after surgery.

Within the scientific community it is evident that lack of physical activity and high calorie intake plays causal roles in the epidemic of obesity and in the development of metabolic disorders. Moreover, understanding the causes that lead to obesity and also its prevention or reversion are of public health priority. This can be managed by either modification of lifestyle (through physical activity to restore energy balance or reduction of calorie intake, proper diet that is rich in fiber, increasing energy outlay) or using adequate medication or alternative techniques. Therefore, to address this crucial subject, different research fields must combine their expertise.

## Author contributions

All authors listed have made a substantial, direct, and intellectual contribution to the work and approved it for publication.
